# Relations of Hospitals with Panel Practitioners

**Published:** 1914-07-11

**Authors:** 


### RELATIONS OF HOSPITALS WITH PANEL PRACTITIONERS

### The London Panel Committee and the Budget Grants.

A meeting of the London Panel Committee on July 7 dealt with the statement in the Budget that grants would be provided for certain public health and health insurance services, including the establishment of centres for the co-operation of practitioners on the panel. The Panel Service Sub-Committee invited the Committee to express the following opinions:—

(1) That in London the proposed centres for the co-operation of practitioners on the panel should be sufficiently numerous, and should be so situated that each practitioner on the panel might have an opportunity, if he so desires, of seeing his industrial patients at such a centre.

(2) That the centre should provide consulting-rooms for a number of practitioners and accommodation for a trained nurse, an *x*-ray operator, a clerk, and, if possible, a chemist on the list of persons supplying drugs and appliances.

(3) That arrangements should be made for the occasional attendance at the centre of consultants.

(4) That the centres should be under the joint control of the Insurance and Panel Committees.

Dr. J. A. Butler criticised the proposals on the ground that they would be the forerunners of a State medical service, and would be unjust to doctors who had provided adequate waiting-room and surgery accommodation. Neither did he think centres would be as convenient as surgeries to the patients.

Dr. T. F. Keenan thought something on the lines of the resolutions was bound to come. The general practitioner in many districts was badly served by the hospitals; the large hospitals should be converted into smaller local establishments which could be distributed throughout London.

Dr. A. Welply reminded the meeting that it was not for it to discuss the advisability of centres for co-operation; they actually formed part of the Budget, and it was necessary that panel practitioners should influence the Government in the details of its proposals.

Dr. Lauriston E. Shaw also pressed this point. He remarked that the Cabinet would be deciding almost immediately how the money would be spent, and if panel practitioners did not make a suggestion, it would be left to those who were so fond of a State medical service to impress their views on the Government. There were two schools of thought in this matter. One school held that the general practitioner should be regarded as the basis to which should be added the specialists; the other school thought the hospitals were the basis of a medical service, and when cases were no longer interesting to the hospitals they should be thrown away to the general practitioner. Members of the Panel Committee would agree with him that the general practitioner, who came first into touch with disease, was the first line of defence, and if so, let them make certain that he was doing his work as well as possible. The general practitioner would find himself in difficulties if he were too isolated. He thought the resolutions propounded the best way of dealing with the question. The other suggestion was that the great hospitals should be given money to start outpost hospitals to which they would send clinical practitioners, and from whom panel practitioners would be able to get a second opinion. Panel practitioners would be very unwise if they allowed the holders of that view to get the first shot and led the Government to think that they did not want the money. Differences in the size of practitioners' lists and variations in local conditions should not be allowed to create disagreements which might prevent the money from coming to panel practitioners as a whole.

Dr. G. B. Batten proposed, and Dr. R. J. Farman seconded, an amendment, which would have the effect of omitting all details and only expressing the Committee's approval of the principle of centres of co-operation.

Dr. J. J. Atteridge thought the meeting must go into details. If the centres were restricted to industrial patients, he feared it might lead to the State sending salaried doctors to see industrial patients at centres, instead of leaving the work to the panel practitioners. In other respects he approved the resolutions.

After further discussion it was agreed that it might be unwise to include a chemist in the staff of the centres, and the clause to this effect was struck out, as was the clause limiting the centres to industrial patients. With these variations the resolutions were adopted.

The Committee passed resolutions describing the last reason given by the London Insurance Committee for not distributing the unallotted funds among practitioners on the panel as a frivolous one, and appointing a deputation to interview the Insurance Commissioners and approach the Government on the question.

Considerable discussion took place on reports dealing with excessive prescribing. The meeting showed itself unwilling to pass resolutions limiting practitioners in the matter of prescriptions, but Dr. B. A. Richmond (the Secretary) urged that money was being squandered through doctors ordering drugs with fancy names, and Dr. H. H. Mills declared there was evidence of wholesale waste on the part of some doctors. Instances were given of the prescribing of proprietary preparations, vaccines, musk, and oxygen at a cost of several pounds. The Committee ultimately passed resolutions declaring that proprietary medicines, preparations in the nature of wholly manufactured foods, and known preparations under branded names should not be supplied at the cost of the Drug Fund.

